# Cultivating Strengths: Extracurricular Activity Participation in Relation to Psychological Wellbeing and Social Functioning in Adolescents with and without ADHD

**DOI:** 10.1007/s10802-026-01458-7

**Published:** 2026-04-11

**Authors:** C. Danielle Green, Elizabeth S. M. Chan, Melissa R. Dvorsky, Olivia R. Baron, Stephen P. Becker

**Affiliations:** 1 Division of Behavioral Medicine and Clinical Psychology, Cincinnati Children’s Hospital Medical Center, 3333 Burnet Avenue, Cincinnati, OH 45229-3039 USA; 2 Department of Pediatrics, University of Cincinnati College of Medicine, Cincinnati, OH USA; 3 Psychology Department, Montclair State University, Montclair, NJ USA; 4 Division of Psychology and Behavioral Health, Children’s National Hospital, Washington, DC USA; 5 Department of Psychiatry and Behavioral Sciences, and Department of Pediatrics, The George Washington University School of Medicine and Health Sciences, Washington, DC USA

**Keywords:** Attention-deficit/hyperactivity disorder, Adolescence, Extracurricular activities, Positive Youth Development, Resilience

## Abstract

Extracurricular activities are viewed as important contexts for supporting adolescent wellbeing and functioning. Yet, less is known about how participation relates to wellbeing across adolescence, particularly among youth at heightened risk for social and emotional difficulties, such as those with attention-deficit/hyperactivity disorder (ADHD). Guided by a Positive Youth Development framework, this study examined associations between overall and domain-specific extracurricular participation and psychosocial outcomes in a racially diverse sample of 3,578 adolescents (ages 15–18; *M* = 15.62), including 588 with ADHD, from the Future of Families and Child Wellbeing Study. Descriptive analyses indicated largely comparable participation frequencies across adolescents with and without ADHD, with modest domain-specific differences. Path models adjusting for demographic covariates indicated that more frequent extracurricular participation was associated with higher wellbeing and greater socially adaptive behavior, unrelated to anxiety symptoms, and was marginally associated with lower depressive symptoms. Domain-specific analyses showed that sports participation was associated with higher wellbeing, socially adaptive behavior, higher anxiety symptoms, and lower depressive symptoms. Volunteering was associated with greater socially adaptive behavior only. Performance participation was associated with lower socially adaptive behavior, and school-based activities with lower wellbeing. Scouting or hobby participation showed mixed associations, including lower wellbeing, socially adaptive behavior, and anxiety, but higher depressive symptoms. Religious participation was unrelated to outcomes. ADHD status rarely moderated associations: however, greater school-based participation was related to lower anxiety among adolescents with ADHD only. Findings suggest extracurricular engagement can support positive functioning, with some structured activities conferring additional emotional benefits for youth with ADHD.

Extracurricular activity participation represents a promising context for adolescent development. Defined broadly as structured and unstructured activities that occur before, during, or after school, these activities provide opportunities for skill-building, social connection, and identity exploration (Eccles et al., 2003). In general adolescent populations, participation has been linked to academic achievement (Eccles et al., 2003), stronger peer relationships (Oberle et al., 2019; Schaefer et al., 2011), higher self-worth (Blomfield & Barber, 2009), and lower engagement in risk behaviors and delinquency (Eccles et al., 2003). Taken together, this body of work points to consistent associations between extracurricular involvement and markers of risk reduction. Nevertheless, most studies have emphasized their role in mitigating negative outcomes, raising important questions about how participation may also contribute to bolstering positive indicators of wellbeing and adaptive functioning.

Beyond whether youth participate in extracurricular activities, the *frequency* of participation has also been identified as a key factor in shaping developmental outcomes. Research suggests that more consistent or intensive involvement amplifies the benefits of extracurricular engagement, including stronger academic achievement, enhanced social skills, and greater school attachment (Mahoney et al., 2006; Vandell et al., 2015). Adolescents who participate frequently in structured activities report lower levels of delinquency and substance use, likely because sustained engagement provides ongoing supervision, structure, and opportunities for skill-building (Eccles et al., 2003; Farb & Matjasko, 2012). However, evidence also cautions against excessive involvement: overscheduled adolescents may experience increased stress and emotional strain (Fredricks, 2012). Thus, the frequency of participation not only shapes the developmental benefits of extracurricular activities but also represents an important factor to consider when evaluating adolescents’ involvement.

Additionally, it is important to differentiate between types of extracurricular activities to better understand whether specific forms of participation are particularly associated with positive development outcomes, as it is unlikely that all activities confer the same advantages or operate through the same mechanisms. For instance, in a representative sample of eleventh grade students across Illinois, Larson et al. (2006) found that participation in sports and performance or fine arts was more strongly associated than other organized activities with experiences that foster initiative, whereas involvement in faith-based groups was more strongly linked to identity exploration and emotion regulation. More recent evidence from the Add Health study involving a nationally representative sample of American students aged 11 to 17 shows contrasting implications of activity type, even after accounting for sex, race, and age. Specifically, youth involved in sports clubs were more likely to commit violent crimes later on compared to their non-participating peers, whereas those engaged in arts-related or academic clubs were less likely to do so (Dain, 2025). Similarly, in a study of extracurricular participation and adolescent alcohol perceptions, school-based activities were associated with strong beliefs about the risks of drinking, whereas faith-based activities conveyed protective effects only when adolescents were highly involved (Szapary et al., 2025). Other work has also highlighted the distinct benefits of activity type: civic engagement activities (e.g., student government, volunteering) have been linked to leadership and prosocial behavior, whereas arts participation supports creativity, identity development, and lower externalizing behaviors (Eccles & Barber, 1999; Fredricks & Eccles, 2006; Zarrett et al., 2009). Taken together, these findings suggest that not all extracurricular contexts are similarly protective and further illustrate the type of activity youth engage in matters not only for the developmental opportunities each setting affords, but also for the social norms and peer contexts they provide. Thus, it is critical to consider type-specific effects when evaluating the broader implications of extracurricular participation.

## Extracurricular Participation in Adolescents with ADHD

Attention-deficit/hyperactivity disorder (ADHD) is a common neurodevelopmental disorder in childhood and adolescence, characterized by developmentally inappropriate levels of inattention, hyperactivity, and impulsivity (American Psychiatric Association, 2013). Beyond core attentional and behavioral symptoms, adolescents with ADHD frequently experience difficulties in peer relationships, emotion regulation, and academic functioning, including elevated rates of peer rejection, social skills deficits, academic underachievement, and negative feedback from adults and classmates (Hoza et al., 2005; Kofler et al., 2017; Sonuga-Barke et al., 2023). Over time, these chronic interpersonal and academic stressors may accumulate, contributing to heightened vulnerability to internalizing difficulties, as repeated experiences of failure, stigma, and social exclusion can undermine self-esteem and increase risk for anxiety and depressive symptoms (Becker et al., 2012; Lee et al., 2016).

Adolescence represents a particularly sensitive developmental period marked by increasing executive functioning demands, identity exploration, and more complex peer relationships (Hinshaw & Becker, 2020). For youth with ADHD, these normative challenges often intersect with existing self-regulation and social difficulties, placing them at elevated risk for peer conflict, school disengagement, and internalizing symptoms during this stage (Lee et al., 2016). Given the cumulative nature of ADHD-related impairments, there is a pressing need to identify contexts that promote resilience and support positive adjustment during this critical developmental window.

Much of the research on ADHD has historically focused on identifying risk factors and impairment, with far less attention paid to protective or promotive factor (Dvorsky & Becker, 2025). Increasingly, researchers and clinicians, as well as individuals with lived experience in ADHD, have emphasized the importance of moving beyond a deficit-oriented lens to examine strengths-based and ecological contributors, which foster adaptation in context of challenges associated with the diagnosis (Dvorsky & Langberg, 2016; Nordby et al., 2023). The Positive Youth Development (PYD) framework is a youth-driven framework that underscores the value of nurturing strengths, providing enriching environments that can facilitate thriving among all youth (Lerner et al., 2012), and has been applied to children and adolescents with ADHD (Chan et al., 2022; Holt, 2016; Shek et al., 2019). Using a PYD framework, the focus shifts from not only reducing impairment but also identifying ways to actively support thriving.

Within this framework, extracurricular activities may be especially meaningful for adolescents with ADHD because they provide structured yet flexible environments that directly target core areas of vulnerability, including social competence, emotion regulation, and opportunities for positive reinforcement. Whereas repeated academic challenges and negative feedback in classroom settings can erode self-efficacy and contribute to disengagement (Hoza et al., 2005; Sonuga-Barke et al., 2023), extracurricular contexts may allow adolescents to engage around shared interests, experience success in non-academic domains, and receive affirmation for strengths such as creativity, leadership, or athletic ability. These environments may also offer naturalistic opportunities to practice self-regulation and social skills in settings that are less evaluative and more intrinsically motivating. Thus, frequent participation may support wellbeing by providing alternative pathways for competence-building and belonging that are not always accessible within traditional academic contexts.

Despite its potential relevance, extracurricular participation has received limited empirical attention in the context of ADHD. Existing research consists primarily of two studies that have compared youth with and without ADHD, generally finding lower levels of activity involvement among those with ADHD (Malek et al., 2025; Wiggs et al., 2023). In a sample of 200 children aged 7–11 years old (*N* = 100 with ADHD), Malek and colleagues (2025) showed that children with ADHD had notably lower participation rates in all types and domains of activities. Wiggs et al. (2023) reported similar patterns in a community sample of 302 adolescents (ages 12–15), with lower rates of participation in both school-based and community activities among adolescents with ADHD compared to their peers without ADHD. Although such between-group comparisons underscore that extracurricular participation may differ for youth with ADHD, they offer little insight into *how* participation may be beneficial, or under what conditions it supports development within this population.

Parents consistently express interest in identifying activities that could support their child’s functioning (Kremer-Sadlik et al., 2010), highlighting a disconnect between family needs and available guidance. Importantly, prior work has focused primarily on exercise and organized sports, with some evidence from community-based samples that consistent participation predicts reductions in ADHD symptoms over time when symptoms are assessed dimensionally rather than by diagnostic status (Pagani et al., 2020). While informative, these studies largely emphasize symptom outcomes and group differences rather than exploring the potential promotive or protective effects of extracurricular participation for youth with ADHD themselves.

## Current Study

Important gaps remain in understanding the role of extracurricular participation for adolescents with and without ADHD. First, little is known about the role of frequency of involvement for adolescents, particularly for subgroups such as adolescents with ADHD. To date, only one study has examined this latter group, focusing narrowly on ADHD symptom reduction, and found that consistent involvement (e.g., at least once a week for the past 12 months) was associated with fewer ADHD symptoms for girls only (Pagani et al., 2020). Although greater participation has been linked to better outcomes in general adolescent samples, it is unknown whether similar patterns apply to youth with ADHD, who may be especially vulnerable to attentional fatigue or difficulties sustaining engagement in extracurricular activities. Second, there is a need to examine the types of extracurricular activities that may differentially support adjustment. Sports, for example, may provide opportunities to practice teamwork and discipline, arts activities may foster creativity and self-expression, religious involvement may offer belonging and moral grounding, and volunteer experiences may promote prosocial identity and community connection. Yet no studies have disaggregated extracurricular activities to evaluate whether these distinctions differ for youth with and without ADHD.

The current study aims to start addressing these gaps by examining extracurricular participation in a large, racially diverse sample of adolescents with and without ADHD. Guided by a Positive Youth Development framework, we investigated extracurricular involvement as a potential promotive context for adolescent wellbeing and adaptive functioning. Specifically, we aimed to: (1) describe patterns of extracurricular participation, including frequency and activity domains, and to examine differences by ADHD diagnostic status; and (2) examine associations between extracurricular participation and adolescent wellbeing, socially adaptive behavior, and internalizing symptoms. Within this second aim, we assessed whether (a) overall participation frequency and (b) specific activity domains were uniquely related to outcomes, and whether these associations differed by ADHD diagnostic status. By adopting a strengths-based lens, this study seeks to advance understanding of how extracurricular contexts may serve as developmental assets for adolescents broadly, while also considering implications for youth with ADHD. Moreover, given well-documented racial and ethnic inequities in access to high-quality extracurricular opportunities (Pearce & Larson, 2006) driven by financial barriers as well as structural factors including disparities in school and neighborhood resources, inclusion of race/ethnicity as a covariate is warranted to account for potential confounding influences on extracurricular opportunities.

### Methods

#### Participant Characteristics

The present study used data from the sixth wave of the *Future of Families and Child Wellbeing Study* (FFCWS; McLanahan et al., 2011), a longitudinal birth cohort study of 4,898 children born in large U.S. cities between 1998 and 2000. The FFCWS employed a stratified, multistage sampling design that oversampled births to unmarried mothers at a 3:1 ratio. The FFCWS sample is racially, ethnically, and socioeconomically diverse, including a substantial proportion of families from underrepresented and low-income backgrounds, providing a rich context for examining developmental and family processes across varied populations. For the current analysis, adolescents were included if they fully completed the Year 15 follow-up assessment and had a prior diagnosis of ADHD as reported by their caregiver. The final analytic sample included 3,578 adolescents aged 15–18 years (M = 15.62). Participants were 51.8% male and racially/ethnically diverse: 49.0% identified as Black, non-Hispanic; 24.9% as Latino/a/e; 16.4% as White, non-Hispanic; 5.4% as multiracial, non-Hispanic; and 2.6% as other, non-Hispanic. Twenty-seven adolescents (0.8%) reported religious affiliation. Mean household income was $60,587.36. Overall, 16.4% of youth (*n* = 588) had a diagnosis of ADHD, and 6.6% were currently taking ADHD medication.

## Measures

**ADHD diagnosis.** Caregivers were asked to report (yes/no) whether their child had ever been diagnosed with ADHD by a professional.

**Extracurricular participation**. Adolescents were asked to report how often they participated in extracurricular activities during the previous school year or current school year (depending on when respondents were completing the measure). Activities were broken down into 6 categories: Athletics or sports teams, group performance activities, scouts/hobbies/clubs, school activities, religious activities, and volunteer activities. Responses were rated on a 5-point scale: 0 (*Never*), 1 (*Less than once a month*), 2 (*At least once a month*), 3 (*Once a week*), and 4 (*Several times a week*).

**Adolescent social skills.** Twelve items adapted from the express subscale of the Adaptive Social Behavior Inventory (Hogan et al., 1992) and the assertion scale of the secondary-level parent and teacher forms of the Social Skills Rating System (Gresham & Elliott, 2008) were used to assess youths’ social skills. Youth responded 1 (*Not true*), 2 (*Sometimes true*), or 3 (*Often true*) to each item. Example items included “I understand others’ feelings like when they are happy, sad, or mad”, “I join group activities without being told to”, and “I start conversations rather than waiting for others to talk first” (Cronbach’s *α* = 0.76). Items were summed to create a total score, with higher scores reflecting higher social skills.

**Anxiety symptoms**. Anxiety symptomology was assessed using a short 6-item version of the Brief Symptom Inventory (Derogatis, 1993). The anxiety dimension of the scale included questions pertaining to symptoms associated with clinical manifestations of anxiety such as restlessness, nervousness, and tension. Participants were asked to characterize the level of distress they experienced over the past month (four weeks), on a 4-point Likert-like scale with questions anchored from 0 (*strongly disagree*) to 3 (*strongly agree*). Example items from the anxiety scale included: “I feel nervous or shaky inside” and “I feel fearful”. A total score for the BSI was calculated by summing the responses from each item. Anxiety was operationalized as a continuous variable, with higher scores indicating higher anxiety. The BSI showed good reliability within the sample (Cronbach’s α = 0.76) and has been used in a prior adolescent sample (Eugene et al., 2021).

**Depressive symptoms.** Depressive symptoms were assessed using the 5-item short version of the Center for Epidemiologic Studies Depression Scale (CES-D; Radloff, 1977). The CES-D is a commonly used scale that measures depressive symptomology, and has been previously used in diverse, non-clinical samples. The abbreviated 5-item scale has been deemed an improvement over the 20-item scale among culturally- and racially diverse youth (Perreira et al., 2005). The scale uses a 4-point Likert-type scale, with each question anchored from 0 (*strongly disagree*) to 3 (*strongly agree*). Example questions from the short version CES-D include: “I feel life is not worth living” and “I feel I cannot shake off the blues, even with help”. A sum score of the CES-D was calculated using all 5 items to determine the levels of depressive symptoms for each participant, with higher scores indicating higher levels of depressive symptoms. The short version of the CES-D showed good reliability within the present sample (Cronbach’s α = 0.74).

**Wellbeing.** Wellbeing was measured using the EPOCH Scale of Adolescent Wellbeing (Kern et al., 2016). The EPOCH consists of 20 items that assess the following domains of wellbeing: engagement (e.g., “I get completely absorbed in what I am doing”), perseverance (e.g., “I finish whatever I begin”), optimism (e.g., “in uncertain times, I expect the best”), connectedness (e.g., “I have friends that I really care about”), and happiness (e.g., “I feel happy”). The EPOCH model defines engagement as the capacity of adolescents to become focused on their activities and tasks; perseverance as the ability to work hard and pursue their goals to completion; optimism as hopefulness and confidence about the future; connectedness as the sense of having positive and supportive relationships with friends and others; and happiness as being happy, content, and fun loving (Kern et al., 2016). Items on each subscale were assessed using a four-point scale, with each question anchored from 1 (*strongly agree*) to 4 (*strongly disagree*). Items were reverse coded to indicate higher scores reflecting greater wellbeing (Cronbach’s α = 0.78).

**Covariates.** Sex (0 = male, 1 = female), race/ethnicity (dummy coded with Black as the reference group; White, Latinx, Multiracial, and Other [including American Indian or Alaska Native, Asian, and Native Hawaiian or Pacific Islander] coded as separate indicators), family income, and ADHD medication status were included as covariates in all regression models to account for potential demographic and clinical differences in outcomes. Sex, race/ethnicity, and income were selected given prior evidence of disparities in both extracurricular participation and psychosocial functioning among adolescents.

## Data Analytic Plan

Descriptive analyses were conducted in SPSS Version 28. Data were first examined for accuracy, missingness, and outliers. 1,320 participants who did not complete any study demographic or outcome measures at this timepoint were excluded, yielding a final analytic sample of 3,578 adolescents. Descriptive statistics were computed for all study variables, and bivariate correlations were examined to assess associations among extracurricular participation, covariates, and outcomes. All inferential analyses were conducted in Mplus Version 8.11 using robust maximum likelihood estimation (MLR) to account for non-normality and to accommodate missing data via full information maximum likelihood (missing data was ~ 5% across study variables). Four continuous outcomes were examined: youth wellbeing, socially adaptive behavior, anxiety symptoms, and depressive symptoms.

Analyses were organized around two primary aims. Aim 1 focused on describing patterns of extracurricular participation, including overall participation frequency and participation across activity domains, as well as differences by ADHD diagnostic status. These questions were addressed using descriptive statistics and bivariate correlations.

Aim 2 examined associations between extracurricular participation (frequency) and youth outcomes using multivariate path models. Across models, outcomes were regressed on extracurricular activity (EA) variables, ADHD diagnostic status (0 = no ADHD, 1 = ADHD), EA × ADHD interaction terms, and covariates (youth sex, household income, ADHD medication status, and race/ethnicity indicators [White, Latinx, Multiracial, Other; Black as the reference group]). Standardized and unstandardized coefficients with 95% confidence intervals were examined (α = 0.05).

To address aim 2a, overall extracurricular participation was operationalized as a standardized continuous composite reflecting frequency of involvement across all activity types. To address aim 2b, participation was examined separately across six domains (sports, volunteering, school-based, performance, scouting, and religious activities). For each domain, a separate multivariate path model was estimated in which participation frequency was examined in relation to youth wellbeing, socially adaptive behavior, anxiety symptoms, and depressive symptoms. Domain-specific EA variables were treated as continuous indicators of participation frequency. ADHD diagnostic status was tested as a moderator of associations between extracurricular participation and youth outcomes via extracurricular activity × ADHD interaction terms included in each model. If significant interaction effects were observed, simple slopes were planned to examine associations between extracurricular participation and outcomes separately for adolescents with and without ADHD.

## Results

### Correlation Analyses

Correlation results are presented in Table 1. Sex was associated with extracurricular participation patterns, showing that female youth reported lower participation in sports (*r* = −.16, *p* <.001) and higher participation in group performance (*r* =.19, *p* <.001), scouts/hobby clubs (*r* =.05, *p* =.008), and school-based activities (*r* =.06, *p* <.001) relative to male youth. Higher household income was associated with greater extracurricular participation across domains (*rs* = 0.05–0.14, all *ps* < 0.01), higher overall extracurricular engagement (*r* =.07, *p* <.001), and higher socially adaptive behavior (*r* =.13, *p* <.001), as well as lower depressive symptoms (*r* = −.08, *p* <.001). Race/ethnicity showed minimal bivariate associations with youth outcomes, with only a small correlation with depressive symptoms (*r* =.04, *p* =.025). ADHD diagnostic status was negatively correlated with overall extracurricular participation (*r* = −.04, *p* =.016) and socially adaptive behavior (*r* = −.09, *p* <.001) and positively correlated with depressive symptoms (*r* =.07, *p* <.001). Overall extracurricular participation was positively associated with socially adaptive behavior (*r* =.24, *p* <.001) and associated with lower depressive symptoms (*r* = −.08, *p* <.001) and higher anxiety symptoms (*r* =.06, *p* <.001). Anxiety and depressive symptoms were strongly correlated (*r* = −.64, *p* <.001).

**Table 1:** Bivariate Correlations of Study Variables

Variable	1	2	3	4	5	6	7	8	9	10	11	12	13	14	15	16	17	18
1. Sex	--																	
2. Income	− 0.01	--																
3. ADHD med	− 0.21^**^	0.03	--															
4. ADHD status	− 0.18^**^	− 0.03	0.77^**^	--														
5. White	0.01	− 0.24^**^	− 0.04	− 0.01	--													
6. Latinx	− 0.01	− 0.06^**^	0.01	− 0.05^**^	− 0.56^**^	--												
7. Multiracial	− 0.05^**^	0.09^**^	− 0.08^*^	− 0.03	− 0.16^**^	− 0.10^**^	--											
8. Other Race	0.02	0.01	0.01	0.03	− 0.23^**^	− 0.14^**^	− 0.04^*^	--										
9. Social Skills	− 0.02	0.13^**^	− 0.06	− 0.09^**^	− 0.05^**^	− 0.00	− 0.01	0.03	--									
10. Wellbeing	− 0.07^**^	− 0.03	− 0.01	− 0.04^*^	0.10^**^	− 0.01	− 0.02	− 0.02	0.44^**^	--								
11. Anxiety Symptoms	− 0.07^**^	0.05^**^	− 0.10^**^	− 0.10^**^	0.01^*^	− 0.04^*^	0.02	− 0.03	0.24^**^	0.21^**^	--							
12. Depressive Symptoms	0.11^**^	− 0.08^**^	0.02	0.07^**^	− 0.01	0.02	0.00	0.03	− 0.30^**^	− 0.43^**^	− 0.64^**^	--						
13. Total Extracurricular Participation	0.07^**^	0.07^**^	− 0.01	− 0.04^*^	− 0.03	− 0.01	0.05^**^	− 0.01	0.24^**^	0.15^**^	0.06^**^	− 0.08^**^	--					
14. Sports	− 0.16^**^	0.11^**^	0.02	− 0.02	0.02	0.04^*^	0.01	− 0.01	0.19^**^	0.18^**^	0.11^**^	− 0.12^**^	0.53^**^	--				
15. Performing Activities	0.19^**^	0.05^**^	− 0.04	− 0.03	0.01	− 0.03	− 0.01	− 0.01	0.08^**^	0.05^**^	− 0.01	− 0.01	0.55^**^	0.06^**^	--			
16.Scouts/ Hobbies Clubs	0.05^**^	0.09^**^	− 0.01	− 0.03	− 0.05^**^	0.00	0.04^*^	0.00	0.10^**^	0.02	0.00	− 0.00	0.59^**^	0.11^**^	0.21^**^	--		
17. School	0.06^**^	0.11^**^	− 0.09^**^	− 0.03^*^	− 0.01	− 0.02	0.06^**^	0.00	0.12^**^	0.06^**^	0.01	− 0.02	0.62^**^	0.09^**^	0.19^**^	0.39^**^	--	
18. Religious	0.03	0.14^**^	0.02	− 0.02	− 0.06^**^	− 0.01	0.03	0.01	0.15^**^	0.07^**^	0.03	− 0.07^**^	0.59^**^	0.16^**^	0.18^**^	0.20^**^	0.25^**^	--
19. Volunteer	0.03	0.08^**^	− 0.03	− 0.05^**^	0.03	− 0.04^*^	0.04^*^	− 0.01	0.18^**^	0.13^**^	0.04^*^	− 0.05^**^	0.62^**^	0.18^**^	0.19^**^	0.26^**^	0.32^**^	0.31^**^

Sex was coded 0 = male and 1 = female. ADHD status was coded 0 = no ADHD and 1 = ADHD. Due to low response frequencies, participants identifying as American Indian or Alaska Native, Asian, and Native Hawaiian or Pacific Islander were combined into a single category labeled Other. *** *p* <.001. ** *p* <.05. * *p* <.10

## Examining Group Differences in Frequency of Extracurricular Activities Participation

Descriptive analyses indicated broadly similar patterns of extracurricular participation frequency across youth with and without ADHD, with modest domain-specific differences. Frequency of sports participation did not differ by ADHD status, *χ²(*4, *N* = 3414) = 4.02, *p* =.404, with approximately one-third of youth in both groups reporting no participation and the largest proportion in each group participating several times per week. Similarly, frequency of participation in group performance activities, *χ²(*4, *N* = 3418) = 6.90, *p* =.141, scouts or hobby clubs, *χ²(*4, *N* = 3414) = 5.95, *p* =.203, and religious activities, *χ²(*4, *N* = 3410) = 7.32, *p* =.120, did not differ significantly by ADHD status. Differences in frequency of school-based activities approached statistical significance, *χ²(*4, *N* = 3417) = 9.41, *p* =.052, with youth with ADHD somewhat more likely to report no participation. In contrast, frequency of participation in volunteer service activities differed significantly by ADHD status, *χ²(*4, *N* = 3415) = 19.69, *p* <.001, with youth with ADHD more likely to report no participation and less likely to engage at least monthly or more frequently. Overall, patterns of extracurricular activity frequency were largely comparable across groups, with the most pronounced group difference observed for volunteer activities. Actual percentages are presented in Table 2.

**Table 2:** Frequencies of Adolescent Extracurricular Participation

Activity	Frequency	Full Sample	ADHD (%)	No ADHD (%)
Athletics or sports teams	Never	31.9	32.1	31.9
	Less than once a month	4.4	5.1	4.2
	At least once a month	10.9	12.6	10.5
	Once a week	12.1	12.3	12.0
	Several times a week	40.8	37.9	41.4
Group performance activities	Never	59.1	63.1	58.4
	Less than once a month	4.0	2.9	4.2
	At least once a month	8.1	8.6	8.0
	Once a week	8.7	7.4	8.9
	Several times a week	20.1	18.0	20.5
Scouts or hobby clubs	Never	73.6	77.1	72.9
	Less than once a month	3.0	1.8	3.3
	At least once a month	8.2	7.6	8.3
	Once a week	9.3	8.3	9.5
	Several times a week	5.9	5.2	6.0
School activities	Never	63.7	67.7	63.0
	Less than once a month	3.9	4.1	3.9
	At least once a month	11.3	8.3	11.8
	Once a week	12.2	12.8	12.1
	Several times a week	8.8	7.0	9.1
Religious services	Never	60.0	61.6	59.6
	Less than once a month	4.4	3.4	4.6
	At least once a month	10.9	13.2	10.4
	Once a week	17.1	14.8	17.5
	Several times a week	7.6	7.0	7.8
Volunteer service activities	Never	47.0	55.2	45.4
	Less than once a month	8.9	6.5	9.4
	At least once a month	26.6	22.1	27.4
	Once a week	11.4	10.3	11.6
	Several times a week	6.1	5.9	6.1

## Overall Extracurricular Participation

Multivariate path models examined whether overall frequency of extracurricular participation was associated with youth outcomes above and beyond demographic and clinical covariates (sex, race/ethnicity, household income, and ADHD medication status). Results indicated that more frequent extracurricular participation was significantly associated with higher levels of youth wellbeing (*β* = 0.18, *p* <.001) and socially adaptive behavior (*β* = 0.27, *p* <.001; see Table 3) while also being associated with lower depressive symptoms (*β* = −0.08, *p* <. 05).

**Table 3:** Regression Analyses Examining Associations Between Extracurricular Participation Frequency and Youth Outcomes

	Wellbeing			Socially Adaptive Behavior		
	β	SE	*p*	β	SE	*p*
ADHD Status	−0.03	0.06	0.606	−0.04	0.06	0.535
Extracurricular Participation	0.18	0.04	**< 0.001**	0.27	0.04	**< 0.001**
Extracurricular X ADHD	−0.03	0.04	0.440	−0.02	0.04	0.607
	**Depressive Symptoms**			**Anxiety Symptoms**		
	***β***	**SE**	***p***	***β***	**SE**	***p***
ADHD Status	0.10	0.05	0.078	−0.13	0.06	**0.033**
Extracurricular Participation	−0.08	0.04	**0.039**	0.06	0.04	0.155
Extracurricular X ADHD	0.01	0.04	0.854	0.02	0.05	0.630

Values represent standardized path coefficients (β). Models adjusted for sex, race/ethnicity, family income, and ADHD medication status

In contrast, extracurricular participation frequency was not significantly associated with anxiety symptoms (*β* = 0.06, *p* =.155). ADHD diagnostic status was only associated with anxiety symptoms (*β* = −0.13, *p* <.05). Neither ADHD diagnostic status nor the extracurricular participation × ADHD interaction significantly was associated with any other outcomes (all *ps* ≥ 0.39), indicating that associations between extracurricular participation and youth outcomes did not differ by ADHD status.

### Domain-Specific Extracurricular Participation

Multivariate path models examined associations between sports, religious, performance, school-based, volunteering, and scouting participation and youth outcomes, adjusting for demographic and clinical covariates (see Table 4). Greater frequency of sports participation was associated with higher youth wellbeing (*β* = 0.18, *p* <.001) and greater socially adaptive behavior (*β* = 0.11, *p* <.001). Sports participation was also associated with fewer depressive symptoms (β = −0.12, *p* <.001), but higher anxiety symptoms (*β* = 0.14, *p* <.001). Religious participation was not significantly associated with wellbeing, socially adaptive behavior, anxiety symptoms, or depressive symptoms (all *ps* > 0.46). Performance participation was associated with lower socially adaptive behavior (*β* = −0.10, *p* =.001), but was not significantly associated with wellbeing, anxiety, or depressive symptoms (all *ps* > 0.08). School-based participation was associated with lower wellbeing (*β* = −0.08, *p* =.025), but was not significantly related to socially adaptive behavior, anxiety symptoms, and depressive symptoms (*ps* > 0.14). However, a significant interaction emerged between school-based participation and ADHD status in predicting anxiety (*β* = −0.07, *p* =.010), indicating that the association between school participation and anxiety differed as a function of ADHD status. Simple slopes analyses indicated that school participation was not significantly associated with anxiety among adolescents without ADHD (*β* = −0.05, *p* =.256, but was significantly associated with lower anxiety among adolescents with ADHD (*β* = −0.11, *p* =.041).Volunteering participation was associated with greater socially adaptive behavior (*β* = 0.10, *p* =.005) and marginally associated with higher wellbeing (*β* = 0.07, *p* =.058), but was not significantly associated with anxiety or depressive symptoms (all *ps* > 0.06). Finally, greater scouting or hobby participation was associated with lower wellbeing (*β* = −0.16, *p* <.001), lower socially adaptive behavior (*β* = −0.10, *p* =.002), lower anxiety symptoms (*β* =.−0.09, *p* =.024), and higher depressive symptoms (*β* = 0.12, *p* =.002). Interaction terms between ADHD status and sports, religious, performance, volunteering, and scouting participation were not significant (*ps* > 0.17).

**Table 4:** Regression Analyses Extracurricular Participation Type Predicting Wellbeing,Socially Adaptive Behavior,Depressive Symptoms,and Anxiety Symptoms

Predictor	Wellbeing			Socially Adaptive Behavior			Depressive Symptoms			Anxiety Symptoms		
	β	SE	*p*	β	SE	*p*	β	SE	*p*	β	SE	*p*
Sports	0.18	0.03	**< 0.001**	0.11	0.03	**< 0.001**	−0.12	0.03	**< 0.001**	0.14	0.03	**< 0.001**
Sports x ADHD	−0.01	0.03	0.653	0.01	0.03	0.069	0.01	0.03	0.823	0.02	0.03	0.491
Performance	−0.06	0.03	0.078	−0.10	0.03	**0.001**	0.04	0.04	0.209	−0.06	0.04	0.130
Performance x ADHD	0.02	0.02	0.458	0.01	0.02	0.579	0.00	0.03	0.966	−0.01	0.03	0.760
Scouts/hobbies	−0.16	0.04	**< 0.001**	−0.10	0.03	**0.002**	0.12	0.04	**0.002**	−0.09	0.04	**0.024**
Scout/hobbies x ADHD	0.01	0.02	0.837	−0.02	0.03	0.459	−0.01	0.02	0.755	0.01	0.03	0.742
School	−0.08	0.04	**0.025**	−0.05	0.03	0.141	0.07	0.04	0.081	−0.05	0.04	0.256
School x ADHD	−0.01	0.02	0.559	−0.03	0.02	0.196	−0.04	0.02	0.131	−0.07	0.03	**0.010**
Religious	−0.03	0.04	0.462	0.00	0.03	0.866	−0.06	0.04	0.089	−0.01	0.04	0.838
Religious X ADHD	0.00	0.02	0.979	0.01	0.02	0.779	0.00	0.02	0.840	−0.00	0.03	0.881
Volunteer	0.07	0.04	0.058	0.10	0.04	**0.005**	−0.01	0.04	0.908	0.01	0.04	0.764
Volunteer X ADHD	−0.01	0.02	0.830	−0.01	0.02	0.830	0.02	0.03	0.500	−0.04	0.03	0.168

Values represent standardized path coefficients (β). Models adjusted for sex, race/ethnicity, family income, and ADHD medication status. ADHD status was included as a predictor in the models but was not significantly associated with outcomes and is not displayed for brevity

## Discussion

The present study is among the first to examine both overall and domain-specific extracurricular participation in relation to wellbeing and psychosocial functioning among adolescents with and without ADHD. Findings highlight substantial heterogeneity across activity contexts, underscoring that increased engagement is not uniformly promotive. Greater frequency of sports participation was associated with higher youth wellbeing and socially adaptive behavior, as well as lower depressive symptoms, but also higher anxiety symptoms. More frequent volunteering was associated with greater socially adaptive behavior only, whereas greater frequency of performance activities was associated with lower socially adaptive behavior. Greater frequency of school-based extracurricular involvement was associated with lower wellbeing. In contrast, greater scouting or hobby participation demonstrated a mixed pattern of associations, including lower wellbeing, socially adaptive behavior and anxiety symptoms, and higher depressive symptoms. Greater frequency of religious involvement was not significantly associated with youth outcomes. Notably, ADHD diagnostic status did not moderate most associations between extracurricular participation and psychosocial outcomes; however, a single interaction indicated that greater school-based participation was associated with lower anxiety among adolescents with ADHD but not among those without ADHD.

### Patterns of Extracurricular Participation

Descriptive analyses indicated that adolescents with and without ADHD reported largely similar frequencies of extracurricular participation across most activity domains. In contrast to prior studies focused on younger samples (Malek et al., 2025; Wiggs et al., 2023), youth with ADHD in the current study were not consistently less engaged in sports, performance activities, scouting, or religious involvement. Differences by ADHD status were modest and domain-specific, with the most pronounced disparity observed for volunteer service activities, where adolescents with ADHD were more likely to report no participation and less likely to engage regularly. This pattern may reflect structural or organizational demands unique to volunteer contexts, such as greater reliance on self-initiation, planning, or adult gatekeeping, which may pose greater challenges for youth with ADHD. Importantly, the overall similarity in participation frequency across groups provides important context for the largely comparable associations observed between extracurricular involvement and youth outcomes by ADHD status.

An important consideration when interpreting these findings is the developmental stage of the current sample. Prior studies reporting lower extracurricular participation among youth with ADHD have largely focused on children ages 7–11 (Malek et al., 2025) and early adolescents ages 12–15 (Wiggs et al., 2023), developmental periods during which activity involvement is more strongly parent-directed and sustained engagement may be shaped by behavioral challenges, school-based constraints, or adult gatekeeping. In contrast, the present study focused on mid-to-late adolescents (ages 15–18), a developmental period characterized by greater autonomy in activity selection and increased alignment between extracurricular involvement and youths’ interests. It is therefore plausible that adolescents with ADHD who remain engaged in extracurricular activities by this stage represent youth who have identified contexts that provide a better person–environment fit, resulting in participation frequencies that more closely resemble those of their peers without ADHD. Together, these findings suggest that ADHD-related disparities in extracurricular participation may attenuate across adolescence as youth gain greater agency in selecting and sustaining involvement in contexts that meet their developmental needs.

### Frequency of Extracurricular Involvement and Wellbeing

Greater frequency of total extracurricular involvement was associated with higher psychological wellbeing and greater socially adaptive behavior across adolescents, including youth with ADHD. These findings align with research in general adolescent populations, where frequent participation has been linked to better self-esteem, academic success, and social competence (Denault & Poulin,^ 2009^; Fredricks & Eccles, 2006). From a Positive Youth Development perspective, regular engagement may be especially important because it creates sustained opportunities for skill-building, consistent interactions with peers and adults, and ongoing exploration of identity. The benefits of frequency may also reflect the accumulation of developmental assets over time, as repeated exposure to supportive contexts can strengthen self-regulatory capacities, enhance a sense of belonging, and reinforce prosocial norms (Berger et al., 2020). Associations between extracurricular participation did not differ by ADHD status, suggesting that frequent engagement may support positive functioning similarly for youth with and without ADHD. Together, these findings extend prior work by demonstrating that frequency of participation, beyond mere involvement, relates to wellbeing and socially adaptive behavior in a large, racially diverse adolescent sample.

In contrast, overall extracurricular participation was associated with lower depressive symptoms and was not associated with anxiety symptoms. On one hand, this pattern is consistent with prior studies showing that extracurricular involvement is often linked with lower depressive symptoms (Abraczinskas et al., 2016; Bohnert et al., 2008; Mason et al., 2009; McHale et al., 2001; Metsäpelto et al., 2014). However, the lack of association with anxiety symptoms suggests that participation alone may be insufficient to buffer against internal distress. Such effects may depend on factors not captured in the current study, including the quality of participation, the social climate within activities, or the degree of fit between activity demands and adolescents’ needs. Additionally, because extracurricular activities are highly social contexts, youth who experience peer difficulties may not derive emotional benefits even when engaged, potentially limiting impacts on internalizing symptoms.

### Domain-Specific Extracurricular Participation and Positive Adaptation

Differential associations across activity domains provide important nuance. Greater frequency of sports participation was associated with higher wellbeing and greater socially adaptive behavior, as well as lower depressive symptoms, but also higher anxiety symptoms, highlighting important tradeoffs associated with increased involvement in athletic contexts. Sports participation may provide structured opportunities for mastery, teamwork, and emotion regulation, all of which are linked to psychological wellbeing (Fredricks & Eccles, 2006a). At the same time, greater intensity of sports involvement may introduce performance pressure and time demands that contribute to elevated anxiety (Beenen et al., 2025). More frequent volunteering was associated with greater socially adaptive behavior but was not related to wellbeing or internalizing symptoms. Volunteer activities may foster purpose, self-efficacy, and social connectedness by promoting civic engagement and facilitating integration into broader social networks (Flanagan et al.,^ 2015^; Youniss & Yates, 1997). The observed benefits of volunteer engagement may be especially salient in mid-to-late adolescence (ages 15–18), a developmental period characterized by a growing orientation toward purpose and contribution beyond the self (Malin et al., 2014). Volunteer activities during this period may be especially salient because they offer opportunities to experience meaningful roles and social impact. Younger children may derive fewer wellbeing benefits from volunteering, as participation at earlier developmental stages is often more parent-directed and less connected to youths’ own values or sense of agency. Thus, associations between volunteer engagement and wellbeing may strengthen across adolescence as youth gain greater choice in activity involvement and are better able to internalize the social and personal significance of service experiences.

In contrast, greater frequency of school-based extracurricular involvement was associated with lower wellbeing, and greater frequency of performance activities was associated with lower socially adaptive behavior. These findings may be understood through a person–environment fit perspective (Midgley et al., 2014), which emphasizes that developmental outcomes depend on the alignment between adolescents’ needs and the demands of their environments. School-based performance activities may involve evaluation, comparison, and public performance, which may heighten stress or self-consciousness and undermine wellbeing, particularly when expectations exceed adolescents’ regulatory or attentional capacities. Prior work indicates that adolescents may experience increased pressure and negative feedback in evaluative contexts (Mueller et al., 2012), and that upward social comparisons in such settings can negatively affect self-concept (Gerber et al., 2018). Although effect sizes were modest, these results highlight that not all extracurricular contexts are uniformly promotive.

Scouting involvement demonstrated a complex pattern of associations across youth outcomes. Greater frequency of participation was associated with lower wellbeing and socially adaptive behavior, as well as lower anxiety symptoms, but higher depressive symptoms. One possible interpretation is that highly structured, adult-directed activity contexts may reduce uncertainty and situational stress, which could contribute to lower anxiety, while simultaneously limiting opportunities for autonomy and peer-driven interaction that support wellbeing and social functioning in adolescence. When such misalignment occurs, structured contexts may support certain aspects of adjustment while constraining others. It is also possible that selection effects contribute to these associations, such that adolescents experiencing greater difficulties may be more likely to enroll in or remain in these types of activities. Finally, greater frequency of religious involvement was largely unrelated to youth outcomes. This pattern may reflect heterogeneity in religious experiences, ranging from supportive community engagement to obligatory participation, and suggests that participation frequency alone may be insufficient to capture the developmental significance of religious involvement.

### ADHD Status as a Moderator of Extracurricular Participation Effects

Contrary to expectations, ADHD diagnostic status did not moderate most associations between extracurricular participation and youth outcomes across either overall frequency or domain-specific models. Relations between participation and wellbeing, socially adaptive behavior, and depressive symptoms were generally similar for adolescents with and without ADHD. However, a single significant interaction emerged between school-based extracurricular participation and ADHD status in predicting anxiety symptoms. Specifically, greater school-based participation was associated with lower anxiety among adolescents with ADHD but was not significantly related to anxiety among adolescents without ADHD. Although this isolated effect should be interpreted cautiously, it suggests that certain structured activity contexts may confer particular emotional benefits for youth with ADHD.

One possible explanation is that school-based activities often provide consistent routines, adult support, and clearly defined expectations, which may support self-regulation and predictability for adolescents with ADHD. Alternatively, these settings may offer opportunities for competence and belonging that are less consistently available in academic contexts for youth with ADHD. At the same time, the largely non-significant moderation effects across activity domains indicate that extracurricular participation may function as a generally supportive developmental context rather than a condition-specific protective factor. Taken together, these findings suggest that extracurricular engagement is generally beneficial across youth, while certain contexts may offer additional advantages for adolescents with ADHD.

### Strengths and Limitations

This study offers several notable strengths, including the use of a large, racially and ethnically diverse sample from the Fragile Future of Families and Child Wellbeing Study (McLanahan et al., 2011), which enhances generalizability and allows for the consideration of structural inequities in access to extracurricular opportunities. Assessing multiple domains of functioning, such as wellbeing, social skills, and internalizing symptoms, further aligns with strengths-based approaches like the PYD framework (Lerner et al., 2012). In so doing, this study moves beyond deficit-focused approaches that are prominent in ADHD research. At the same time, several limitations warrant attention. The cross-sectional and observational nature of the study precludes causal inference or directionality of associations, and longitudinal designs are needed to clarify direction of effects. Future research using intervention designs or causal inference approaches (e.g., quasi-experimental methods, propensity score matching, or randomized trials of extracurricular programming) will be critical for testing whether extracurricular participation exerts causal effects on adolescent wellbeing. Further, reliance on adolescent self-report may introduce bias, though adolescents are often considered the most reliable reporters of internal states such as mood, anxiety, and wellbeing (De Los Reyes & Kazdin, 2005). Incorporating multiple informants in future research would reduce mono-informant bias. Further, future research should examine differential effects by race or ethnicity, taking into account unique barriers and strengths that may influence how youth with ADHD benefit from each type of extracurricular activity.

Although the study adjusted for key demographic and contextual covariates, the present study lacked detailed assessments of ADHD symptom dimensions, co-occurring psychiatric or neurodevelopmental conditions, and activity quality or structure, all of which likely shape how adolescents engage with and benefit from extracurricular contexts. Additional individual level factors that may influence the selection of extracurricular activities include our correlational finding that girls with ADHD reported higher depression, lower wellbeing, and lower social skills than boys with ADHD. This pattern may reflect the higher rates of co-occurring internalizing difficulties in adolescent girls (Gutman & Codiroli McMaster, 2020) and the potential greater violation of social norms associated with ADHD symptoms (Maccoby, 1990; Mikami & Lorenzi, 2011), which may contribute to higher levels of socioemotional difficulties among girls with ADHD relative to boys who may face different developmental expectations. Contextual factors, such as peer climate and adult mentorship which were not assessed, are also critical to consider. While extracurricular domains were examined separately, more granular distinctions within activity categories were unavailable. Accordingly, findings should be interpreted as associative rather than mechanistic. It is also possible that adolescents with higher pre-existing wellbeing or stronger socially adaptive skills are more likely to seek out and sustain participation in extracurricular activities, raising the possibility of selection effects and reverse causation. Longitudinal designs will be essential for disentangling whether extracurricular participation promotes wellbeing, wellbeing facilitates participation, or both processes operate concurrently.

### Future Directions & Clinical Implications

The present findings suggest that extracurricular participation, particularly frequent engagement and involvement in sports and volunteer activities, may be associated with higher wellbeing and stronger socially adaptive behavior across adolescents, including youth with ADHD. In contrast, school-based and performance activities were associated with small decreases in wellbeing, underscoring the importance of examining activity characteristics and person–environment fit. Another critical next step is to better understand the mechanisms underlying these associations. Although many extracurricular activities are structured, structure alone is unlikely to explain domain-specific differences; instead, benefits may depend on how structure is paired with prosocial purpose, opportunities for contribution, and the degree of evaluative pressure embedded within activities. For example, volunteer contexts may uniquely emphasize collective purpose and civic engagement while involving fewer performance-based evaluations, whereas school-based and performance activities often include competition and public evaluation that may attenuate wellbeing benefits. Access to these opportunities is also unevenly distributed, underscoring the need for policies and programs that expand equitable participation in extracurricular contexts linked to positive adjustment.

It remains unclear whether observed effects stem primarily from opportunities for social connection and belonging, experiences of accomplishment and mastery, or combinations of these factors. Unpacking these mechanisms is essential, as it may allow for the development of targeted interventions that maximize the specific elements of extracurricular contexts most beneficial to youth with ADHD. Another important avenue for future research involves examining the long-term effects of extracurricular involvement. As noted above, our cross-sectional design provides only a snapshot in time, leaving open questions about whether benefits are sustained over development. Longitudinal studies are needed to determine whether early participation creates cumulative advantages or “snowball effects” that extend into educational attainment, career success, and life satisfaction. Such work could also clarify whether extracurricular participation buffers against the emergence of more serious mental health difficulties across adolescence and into adulthood. Related, analyzing extracurricular activities while controlling for others, either in blocks or singly, could yield important clinical insights on which extracurricular is more important for positive youth development over and above the others. Finally, future work should focus on identifying the optimal balance of extracurricular participation. Although our results suggest that greater frequency of involvement is linked to higher wellbeing and stronger social functioning, excessive engagement may lead to stress, burnout, or strain on family resources. Importantly, opportunities for frequent participation are not evenly distributed. Structural inequities in access to transportation, program costs, neighborhood resources, and school offerings may constrain how often adolescents can participate, particularly for youth from socioeconomically disadvantaged households or those already navigating the additional demands associated with ADHD. Determining the “right dose” of extracurricular participation will provide families, educators, and clinicians with practical, evidence-based guidance for fostering resilience and thriving among adolescents with ADHD.

## Conclusion

Our results align with the PYD framework (Lerner et al., 2012), which emphasizes building strengths through enriching environments while recognizing that not all activities confer equal benefits. Adolescents vary widely in strengths, challenges, and cultural contexts, making a “one-size-fits-all” recommendation insufficient. Attention to activity quality, fit, frequency, and accessibility can maximize developmental benefits while addressing unique needs. Moreover, understanding how extracurricular participation functions across racially and ethnically diverse populations is critical, as disparities in access and engagement persist (Green & Langberg, 2022). Future research should prioritize inclusive samples to ensure recommendations for promoting thriving among youth are equitable and culturally responsive.

## Data Availability

This research was based on data from the FFCWS that was provided by Princeton University’s Center for Research on Child Wellbeing (CRCW) and the Columbia Population Research Center (CPRC). These data can be found here: https://ffcws.princeton.edu/documentation. Code for any analyses can be obtained from the first author.
